# Herbivory by Striped Stem Borer Triggers Polyamine Accumulation in Host Rice Plants to Promote Its Larval Growth

**DOI:** 10.3390/plants12183249

**Published:** 2023-09-13

**Authors:** Hao Zhang, Chaoyue Gai, Min Shao, Linzhi Fang, Xinyu Li, Yuanyuan Song, Rensen Zeng, Daoqian Chen

**Affiliations:** 1 Key Laboratory of Ministry of Education for Genetics, Breeding and Multiple Utilization of Crops, Key Laboratory of Ministry of Agriculture and Rural Affairs of Biological Breeding for Fujian and Taiwan Crops, College of Agriculture, Fujian Agriculture and Forestry University, Fuzhou 350002, China; 2 Shandong Branch of Sinochem Agriculture Holdings, Zibo 256304, China

**Keywords:** *Oryza sativa*, striped stem borer, polyamine, putrescine

## Abstract

Polyamines (PAs) are ubiquitous low-molecular-weight aliphatic polycations in all living organisms, which are crucial for plant response to abiotic and biotic stresses. The role of PAs in plant disease resistance has been well documented. However, their involvement in plant–pest interactions remains unclear. Here, the role of PAs in rice against striped stem borer (SSB, *Chilo suppressalis* Walker), a destructive pest in rice production worldwide, was investigated. SSB larval infestation led to a substantial accumulation of free putrescine (Put) in rice seedlings, which was in parallel with an elevated expression of host PA biosynthesis genes *Arginine Decarboxylase1* (*ADC1*) and *ADC2*. Moreover, SSB larval oral secretion application with wounding further raised the transcripts of *ADC1* and *ADC2* in rice compared with wounding treatment alone. The larval growth on both rice plants and artificial diet was promoted by the exogenous application of PA and inhibited by a PA biosynthesis inhibitor. On the other hand, the rice defense responses, including polyphenol oxidase (PPO) and peroxidase (POD) activities, as well as protease inhibitor level, were enhanced by a Put supplement and reduced by an ADC inhibitor. Our results indicate that SSB herbivory triggers polyamine accumulation in host rice plants, which is beneficial to SSB in rice–SSB interaction.

## 1. Introduction

Rice striped stem borer (*Chilo suppressalis* Walker, SSB) is a highly destructive pest in all rice (*Oryza sativa* L.) ecosystems and causes enormous yield losses [1,2]. SSB larvae bore into the stem and feed inside, resulting in typical external symptoms such as “dead hearts” at the tillering stage and “whitehead” at the reproduction stage [3]. To date, the only effective method for controlling SSB in regular rice production is the use of chemical pesticides, which not only significantly increases production cost, but also raises insect resistance, environmental toxicity, and concerns for human health [4,5]. Therefore, the demand for innovative and sustainable strategies to control SSB is particularly urgent. However, the mechanism underlying the interaction between rice and SSB remains unclear.

Polyamines (PAs) are low-molecular-weight aliphatic amines that are widely found across all living organisms, including bacteria, animals, and plants [6,7]. PAs are essential for regulation of gene expression, signal transduction, ion-channel function, and DNA and protein synthesis, as well as cell proliferation and differentiation [7,8,9]. In plants, the most common PAs are putrescine (Put), spermidine (Spd), and spermine (Spm). The PA biosynthetic pathway in plants differs from that in animals, which can generate Put from both L-arginine and L-ornithine, whereas in animals, L-ornithine is the sole precursor. Put is generated through the catalytic actions of ornithine decarboxylase (ODC) and arginine decarboxylase (ADC) in three steps in plants. Put subsequently receives an aminopropyl moiety via the catalytic actions of spermidine synthase (SPDS) to produce Spd, which is further transformed into Spm by spermine synthase (SPMS). The aminopropyl moieties required for these reactions are donated by decarboxylated S-adenosylmethionine (dcSAM), a compound synthesized by S-adenosyl-methionine decarboxylase (SAMDC) using S-adenosyl-methionine (SAM) as a substrate. SAMDC is considered as the rate-limiting enzyme in the synthesis of Spd and Spm [10]. Moreover, PA oxidation plays a pivotal role in the regulation of PA homeostasis in plant development and plant response to diverse stress. Copper-containing amine oxidases (CuAOs) are mainly responsible for diamine oxidation, whereas flavin-containing PA oxidases (PAOs) catalyze the oxidative deamination of “higher PAs” [6,11].

The involvement of PAs in plant disease resistance has been well documented [6,11]. The elicitation of PA biosynthesis in response to pathogens of different lifestyles has been extensively reported [6,8]. It was reported that PA levels rise considerably in plant tissues infected by fungi, bacteria, and viruses [12,13,14]. The *adc* loss-of-function mutants and *adc* silenced lines with reduced Put levels are more susceptible to pathogens [15,16]. The exogenous application of Put and overexpression of the *AtADC2* gene induce the expression of plant-defense-related genes and promote the local biosynthesis of salicylic acid [16,17]. It is generally accepted that PA oxidation is critical for plant defense by triggering the hypersensitive response against pathogen attack [6,11,18].

Although the role of plant PAs in response to various microbial pathogens has been well documented, there is limited information on their involvement in plant–pest interactions [19,20]. The elicitation of host plant PA biosynthesis in response to insect herbivorous has been reported in a few plant–pest interactions [19,21,22]. One proposed function of increased PAs in plant defense is the blocking of glutamatergic neuromuscular junctions by phenolic PAs, leading to the paralysis of insect skeletal muscles [21,23]. Contrary to other interactions where PAs play a positive role in resistance, a study on wheat–Hessian-fly (*Mayetiola destructor*) interactions found that the virulent larvae could usurp the wheat PA biosynthesis machinery to obtain their own required nutrient substance [19]. Therefore, the upregulation of PAs in the host plant in response to insect herbivores can benefit both the host plant and insect pest, and the outcome might depend on the type of the pest and host species [6,11].

Given the diverse roles of PAs in plant resistance to pathogens and insect herbivores, the present study aims to examine the role of host PA biosynthesis during rice–SSB interaction. Two primary hypotheses are proposed. First, it is hypothesized that the PA production of host rice will increase in response to SSB larval infestation, based on numerous reports of the elicitation of PA biosynthesis by biotic stresses. Second, it is hypothesized that the elevated PA level will benefit SSB larval growth, as observed in various organisms. In this study, we analyzed the PA levels and the transcript levels of key PA biosynthesis genes in rice plants in response to feeding by SSB larvae. The exogenous Put or its synthesis inhibitor were applied on both rice plants and artificial diet to clarify the role of PAs in rice–SSB interaction. The present study provides insights into the complex interplay between rice and SSB, shedding light on potential strategies for pest management by manipulating plant PA metabolism.

## 2. Results

### 2.1. PA Levels in Response to SSB Larval Infestation

**Figure 1:**
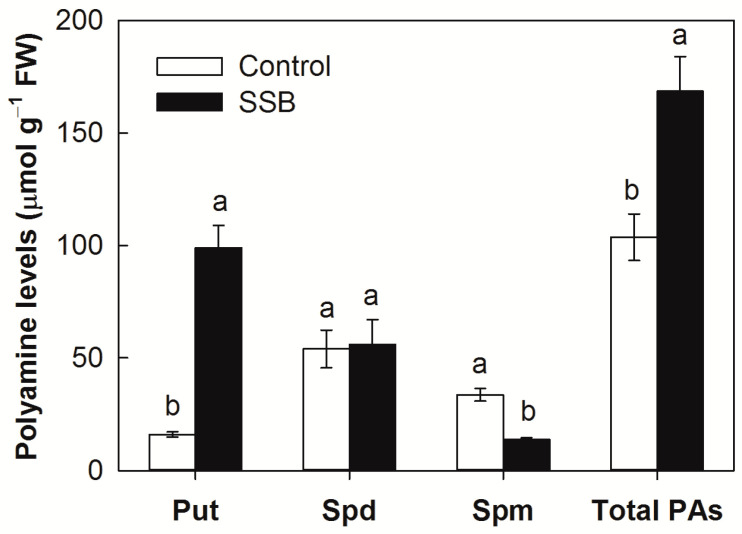
Polyamine (PA) levels in the stems of rice plants attacked by *Chilo suppressalis* (SSB) after 3 days of larval infestation. Put, putrescine; Spd, spermidine; Spm, spermine. Values are means ± SE (n = 4). Different letters above bars indicate statistically significant differences between treatments using the least significant differences (LSD) post hoc test (*p* < 0.05).

Consistent with hypothesis, SSB larval infestation induced a substantial increase of free Put, with the final Put concentration in SSB-infested stem tissues 6.2 times that of uninfected tissues (Figure 1). After SSB larval infestation, the Spd concentration remained unchanged, whereas Spm concentration declined 59.1%. Total free PAs increased about 1.6-fold after SSB larval infestation.

### 2.2. Expression Levels of PA Biosynthesis Genes in Response to SSB Larval Infestation

**Figure 2:**
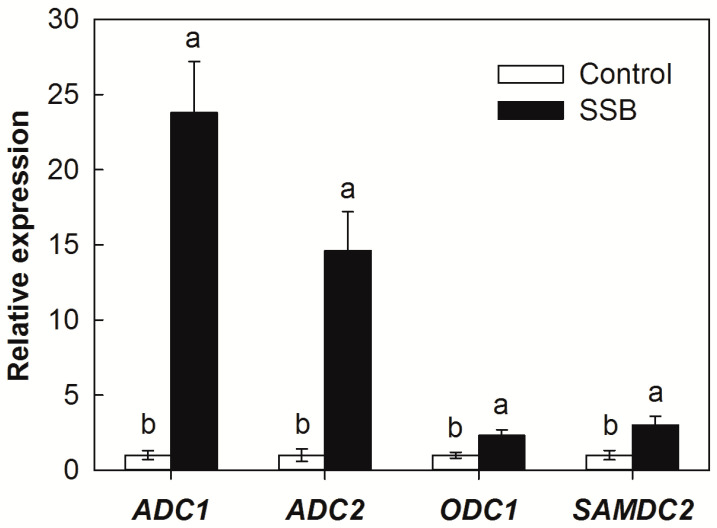
Transcript levels of polyamine biosynthesis genes in the stems of rice plants attacked by *Chilo suppressalis* (SSB) after 3 days of larval infestation. *ADC*, *arginine decarboxylase*; *ODC*, *ornithine decarboxylase*; *SAMDC*, *S-adenosyl-methionine decarboxylase*. Values are means ± SE (n = 4). Different letters above bars indicate statistically significant differences between treatments using the least significant differences (LSD) post hoc test (*p* < 0.05).

To investigate the potential effects of SSB larval infestation on host PA biosynthesis responses, we examined the endogenous transcript levels of *ADC1*, *ADC2*, *ODC1*, and *SAMDC2* in rice plants exposed to SSB larvae infestation. These PA biosynthesis genes were selected because they were significantly induced by SSB larval infestation in previously published RNA-seq data collected from rice plants infested with third-instar SSB larvae for 24 h [24] (Supplementary Table S1). SSB larval infestation triggered the expression of the ADC pathway genes of PA biosynthesis. The mRNA levels of *ADC1* and *ADC2* increased 23.8 and 14.6 times compared with those uninfected (Figure 2). SSB larval infestation also affected the expression level of *ODC1* and *SAMDC2*, but less strongly, with increases of only 2.3 and 3.0 times, respectively.

### 2.3. Effects of SSB Larvae Oral Secretion on the Expression of PA Biosynthesis Genes

**Figure 3:**
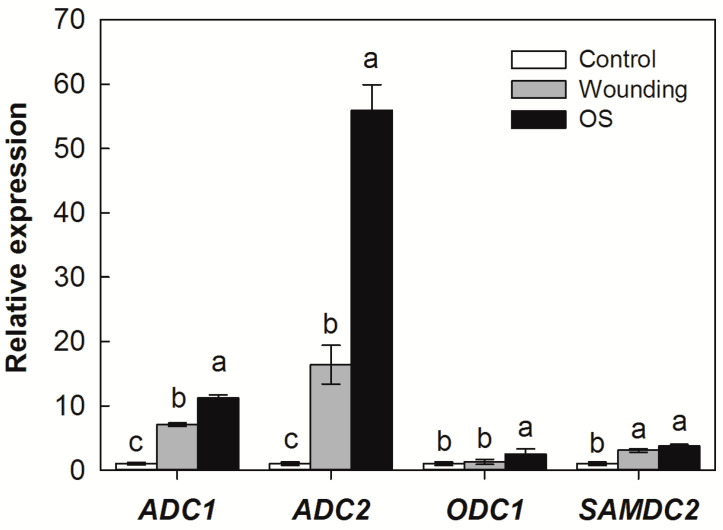
Transcript levels of polyamine biosynthesis genes in wounded rice plants supply with oral secretion (OS) collected from *Chilo suppressalis* (SSB) for 1 h. *ADC*, *arginine decarboxylase*; *ODC*, *ornithine decarboxylase*; *SAMDC*, *S-adenosyl-methionine decarboxylase*. Values are means ± SE (n = 4). Different letters above bars indicate statistically significant differences between treatments using the least significant differences (LSD) post hoc test (*p* < 0.05).

To investigate the potential effects of SSB larvae oral secretion (OS) on host PA biosynthesis responses, we tested the influences of mechanical wounding and OS application on the expression levels of PA biosynthesis genes. Consistent with SSB larval infestation, both mechanical wounding and OS treatment induced the expression of *ADC1* and *ADC2*, but the magnitude of induction was significantly higher in OS-treated plants (Figure 3). The mRNA levels of *ADC1* and *ADC2* increased 7.1- and 16.4-fold in wounded plants, respectively, whereas they increased 11.2- and 56.0-fold in OS-treated plants. Mechanical wounding and OS treatment also affected the expression level of *ODC1* and *SAMDC2*, but less strongly than it affected *ADCs*. There was no significant difference in the induction of *SAMDC2* between mechanical wounding and OS treatment.

### 2.4. Effects of Exogenous Application of PAs and D-Arginine on Larval Performance on Rice Plants

**Figure 4:**
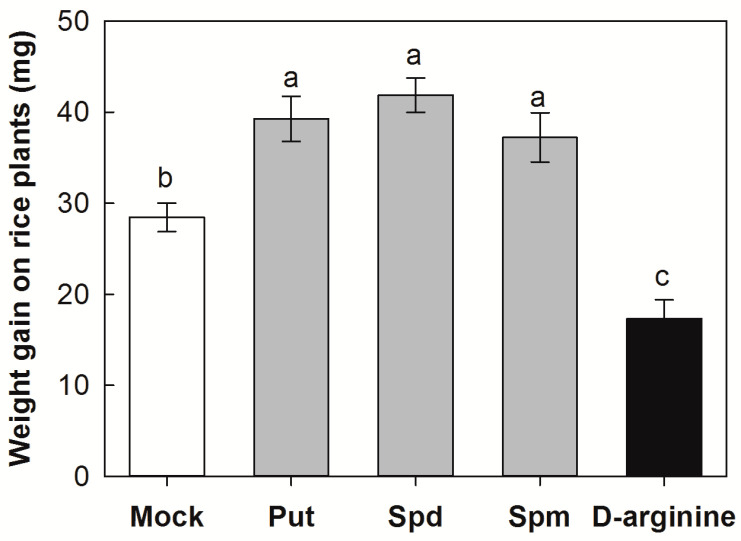
Weight gain of *Chilo suppressalis* (SSB) larvae after 3 days of infestation on rice plants with application of putrescine (Put, 0.5 mM), spermidine (Spd, 0.5 mM), spermine (Spm, 1 mM), and D-arginine (an inhibitor of arginine decarboxylase, 0.5 mM). Mock, blank group without any treatment. Values are means ± SE (n = 20). Different letters above bars indicate statistically significant differences between treatments using the least significant differences (LSD) post hoc test (*p* < 0.05).

The application of Put, Spd, and Spm on rice significantly decreased plant resistance to SSB infestation. The weight gain of SSB larvae fed on mock-treated plants was increased by 28.5 mg 3 d after insect infestation, whereas larvae fed on Put-, Spd-, and Spm-treated plants increased in mass by 38.4, 42.2, and 37.3 mg, respectively (Figure 4). In contrast, the exogenous application of D-arginine, an inhibitor of ADC, significantly increased the resistance of rice plants to SSB. SSB larvae0.85 fed on D-arginine-treated plants increased in mass by only 17.4 mg.

### 2.5. Effects of Exogenous Application of Put and DMFO (Difluoromethylornithine) on SSB Larval Growth on an Artificial Diet

**Figure 5:**
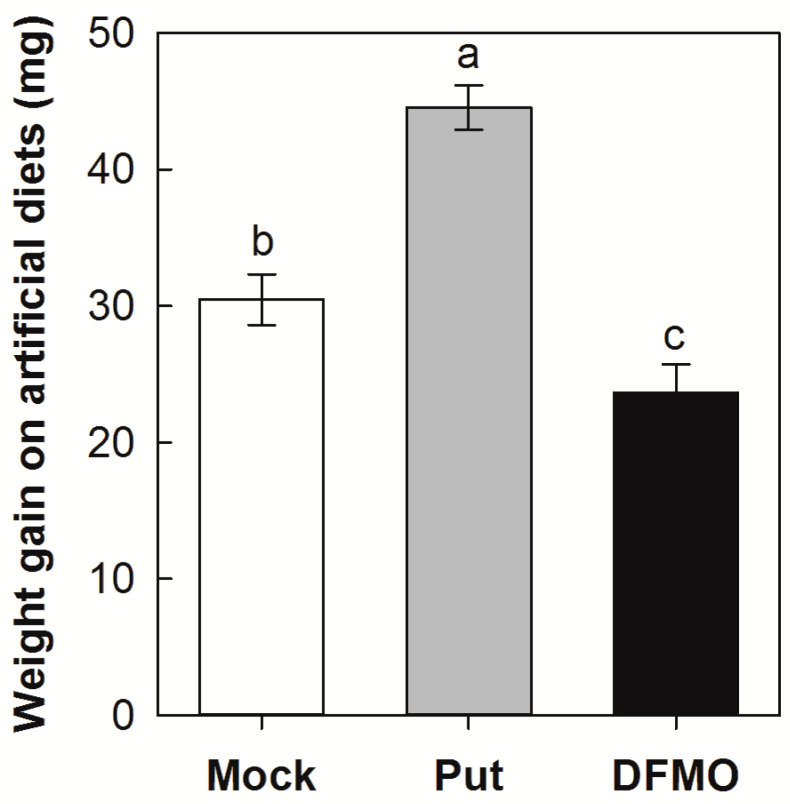
Weight gain of *Chilo suppressalis* (SSB) larvae after 3 days of incubation on artificial diets with application of putrescine (Put, 0.01 mg·g^−1^) and DFMO (difluoromethylornithine, an inhibitor of ornithine decarboxylase, 1 mg·g^−1^). Mock, blank group without any treatment. Values are means ± SE (n = 30). Different letters above bars indicate statistically significant differences between treatments using the least significant differences (LSD) post hoc test (*p* < 0.05).

To further confirm the role of Put in SSB larval growth, we evaluated the influences of the exogenous application of Put and DFMO, an inhibitor of ODC, on SSB larval growth on artificial diets. As shown in Figure 5, Put application significantly increased the SSB larval growth, whereas DFMO application significantly decreased the SSB larval growth. SSB larvae fed on the mock-treated artificial diet increased in mass by 31.3 mg 3 d after incubation, whereas larvae fed on the artificial diet supplied with Put (0.01 mg/g, equivalent to SSB larvae infested stem tissues) and DFMO increased in mass by 44.5 and 24.3 mg, respectively.

### 2.6. Effects of Exogenous Application of Put and D-Arginine on Activities of Defense-Related Enzymes in Rice Plants

**Figure 6:**
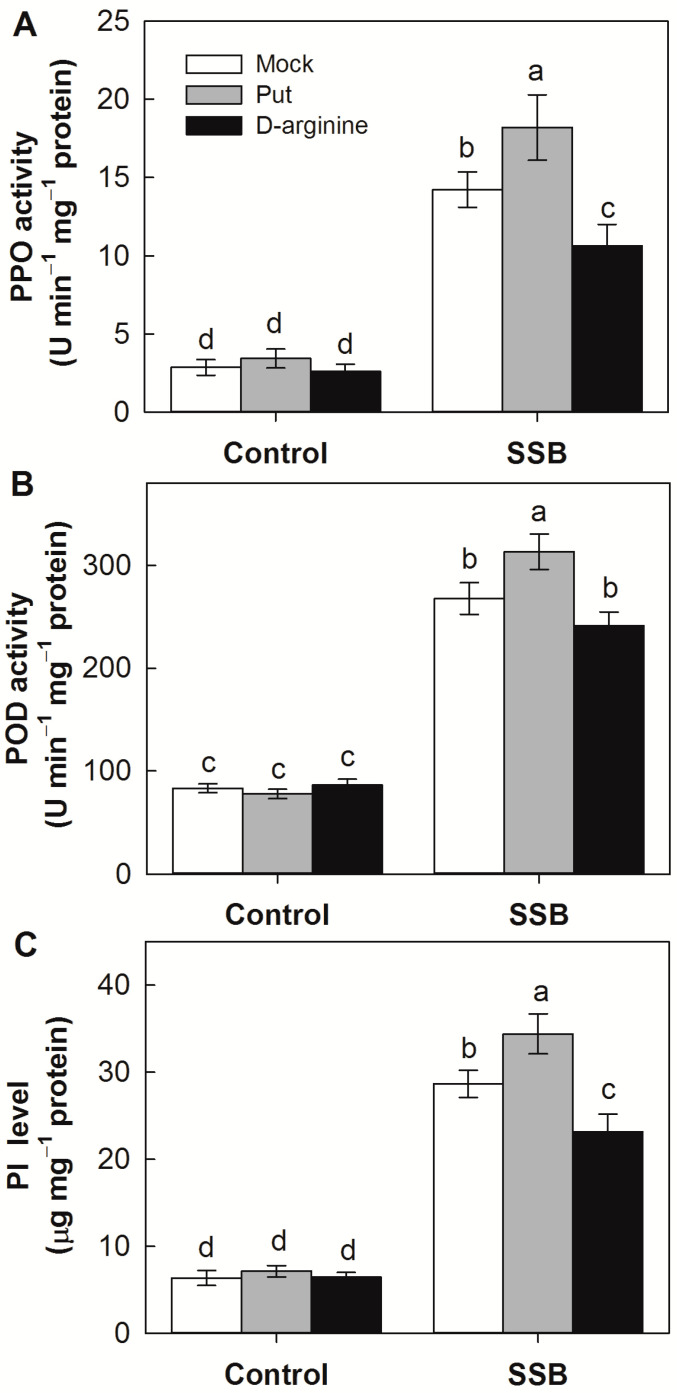
Effects of putrescine (Put, 0.5 mM) and D-arginine (an inhibitor of arginine decarboxylase, 0.5 mM) on activity levels of polyphenol oxidase (PPO, (**A**)), peroxidase (POD, (**B**)), and protease inhibitor (PI, (**C**)) in attacked rice plants by *Chilo suppressalis* (SSB) after 3 days of larval infestation. Mock, blank group without any treatment. Values are means ± SE (n = 4). Different letters above bars indicate statistically significant differences between treatments using the least significant differences (LSD) post hoc test (*p* < 0.05).

To determine the potential impact of PAs on plant antiherbivore defense responses, we examined the influences of the exogenous application of Put and D-arginine on activity levels for polyphenol oxidase (PPO), peroxidase (POD), and protease inhibitor (PI) (Figure 6). Neither Put nor D-arginine application significantly altered PPO, POD, or TrypPI levels in control plants without SSB inoculation. Importantly, PPO, POD, and TrypPI levels were induced in all SSB-attacked plants, but in all cases, the degree of induction was higher in Put-treated plants and lower in D-arginine-treated plants. In response to SSB attack, the activities of POD, PPO, and TrypPI were 27.9%, 16.9%, and 20.1%, respectively, higher in Put-treated plants relative to untreated seedlings. Conversely, the levels of POD, PPO, and TrypPI were 25.2%, 9.8%, and 19.2%, respectively, lower in D-arginine-treated seedlings than those in untreated plants after SSB attack.

## 3. Discussion

Polyamines are essential components found universally in all living organisms, playing a vital role in cell survival [25,26]. In plants, PAs are crucial regulators of growth and development, and their metabolism undergoes substantive changes in response to both biotic and abiotic stresses [9,27,28]. Previous studies have demonstrated substantial increases in all three of the most abundant Pas, Put, Spd, and Spm, following Hessian fly (*Mayetiola destructor*) feeding in susceptible wheat (*Triticum aestivum*) plants [19], whereas the bird cherry-oat aphid (*Rhopalosiphum padi*) feeding on triticale (*Triticosecale*) seedlings at the beginning caused a substantial increase in Spd [29]. In the present study, the SSB larval infestation induced a substantial increase of Put in rice in in parallel with a massive increase of the transcript of *ADCs*, whereas the Spd concentration remained unchanged and the Spm concentration decreased after infestation (Figure 1). These results indicate that SSB feeding triggers Put accumulation in host rice plants and the type of PA elicited by insect herbivory might vary on the different types of the pest and host species. In general, both ODC and ADC pathways in plants mediate polyamine biosynthesis in response to biotic [6] and abiotic stresses [30]. In the case of the wheat–Hessian-fly interaction, ODC-mediated Put biosynthesis played a predominant role in herbivore-induced PA accumulation [19]. The present results show a larger increase in *ADC* transcript levels (up to 23.8-fold for *ADC1* and 14.6-fold for *ADC2*) than *ODC1* transcripts (2.2-fold only) in response to SSB larval infestation, implicating ADC-mediated Put biosynthesis as the predominant entry into this pathway (Figure 2). Therefore, the type of PA biosynthesis pathway induced by insect herbivory might depend on the type of the pest and host species. In the case of rice–SSB interaction, the larval infestation mainly triggers Put accumulation mediated by the ADC pathway in the host plant.

PAs are widely implicated in plant defense or susceptibility during plant interaction with pathogens and pests [31,32]. In the present study, we found that exogenous Put in both rice nutrient solution and artificial diets resulted in a significant increase in larval performance. On the other hand, the experiment utilizing either D-arginine to block rice ADC activity (responsible for conversion of arginine to Put) or DFMO to inhibit the activity of ODC, which converts ornithine to Put in SSB larvae, resulted in a significant decrease in larval growth (Figure 4 and Figure 5). These results provide evidence that SSB-triggered Put production would benefit the SSB larvae themselves. Obligate plant feeders generally commonly employ resource manipulation of the host plant to ensure their sustenance [33,34]. Some gall-forming insects use effector-based mechanisms to reorient the host’s physiology, creating a favorable environment that offers physical protection and high-quality nutrients [35,36]. Similar to amino acids, the PA pool in a living organism is derived from de novo synthesis, dietary supply, or both [36]. Importantly, PAs are growth factors and are required to maintain metabolic processes in all organisms, which are essential for regulation of gene expression, signal transduction, ion-channel function, and DNA and protein synthesis, as well as cell proliferation and differentiation [9,10,11]. Several studies have highlighted the benefits of an exogenous supply of PAs in the diet during insect development [37,38]. It is worth noting that SSB larval OS application enhanced the expression of PA biosynthesis genes further than the mechanical damage (Figure 3). Therefore, it is possible that SSB larvae use an effector-based mechanism to manipulate the PA biosynthesis in the host plant to acquire nutrients for their own growth.

Because PAs provide a degree of protection against pathogen attack and various stresses [6,11], their elevated production might potentially offer benefits to both the insect herbivore and the host plant. Upon attack, plants initiate defense responses involving defense-related enzymes such as PPO and POD, as well as protease inhibitors [39]. The present study also assessed the potential effects of PAs on plant defense responses by the exogenous application of Put and D-arginine to rice plants. It was found that the activities of PPO and POD, as well as the levels of protease inhibitors, were enhanced by exogenous Put and inhibited by the PA biosynthesis inhibitor (Figure 6), suggesting that SSB-triggered Put production would also benefit the host defense responses. Notably, although alteration rice Put, by the exogenous application of Put or D-arginine, impacts rice defense responses, its effect is limited (Figure 6). It has been proposed that the role of PAs in modulating plant defense responses during interactions with various pathogens and pests might depend on the specific PAs involved and their relative abundance [6,8,11]. Transgenic manipulation of PA biosynthetic genes, such as *ADC*, *SAMDC*, *SPDS*, or *SPMS*, often results in the augmented accumulation of PAs and improves the host plant’s resistance against a wide spectrum of pathogens, including fungi and bacteria. However, it is noteworthy that an increase in Put biosynthesis within the host plant, without a proportional conversion of Put into Spd and Spm, can sometimes render the plant more susceptible to fungal pathogens. For instance, in interactions between oat (*Avena sativa* L.) and the powdery mildew pathogen (*Blumeria graminis* f.sp. *avenae*), susceptible oat cultivars tended to accumulate higher levels of Put compared to resistant cultivars during the early infection stage [40]. Conversely, the Spd content was higher in the resistant cultivars compared to the susceptible ones at the same time point, suggesting that the increased production of Spd, but not Put, in the host contributes to resistance against the powdery mildew pathogen. Similarly, in the case of maize–*Aspergillus-flavus* interaction, the resistant maize line exhibited higher levels of Spd and Spm compared to the susceptible line [8]. Hence, the accumulation of Put, rather than Spd or Spm, in rice plants in response to SSB larval infestation may be one potential explanation for why the host defense benefits less from the PA production triggered by SSB. In addition, PA oxidation was proposed as a critical part for PA-mediated plant defense by contributing to the hypersensitive response against pathogen attack. However, no increase in transcripts of rice PA oxidases was observed in this study, possibly due to the differences in plant defense strategies against different pathogens and pests.

In summary, in the case of rice–SSB interaction, the larval infestation triggers Put accumulation, which benefits both the host plant defense responses and insect herbivore larva growth, and the outcome is that SSB benefits more and performs better on rice plants. This study implies that reducing the Put level, particularly through the inhibition of its inductive synthesis upon SSB damage, might be an efficient strategy for breeding SSB-resistant varieties. Further efforts to identify the key factors regulating plant PA metabolism in response to insect herbivores and potential insect effectors manipulating host PA metabolism are promising and critical for improving rice resistance against insect herbivores.

## 4. Materials and Methods

### 4.1. Plant Materials and Growth Conditions

The seeds of rice (*Oryza sativa* L. cv. Nipponbare) were subjected to surface sterilization using 1% (*v*/*v*) sodium hypochlorite (NaClO) for 15 min, followed by rinsing with distilled water four times. Sterilized seeds were then transferred to a seeding tray for germination. After seven days, the uniform healthy seedlings were transplanted into a plastic 5 L box (L × W × H: 35 cm × 25 cm × 12 cm) containing full-strength modified Kimura B nutrient solution, with pH adjusted to 5.6, as described by Chen et al. [41]. The nutrient solution was refreshed every three days. The rice plants were cultivated in a greenhouse under a 12 h/12 h day/night cycle, with the temperature at 27 °C/23 °C, a relative humidity of 75%, and natural sunlight for an additional 30 days.

### 4.2. Herbivore Treatment

The laboratory-maintained rice striped stem borer (*Chilo suppressalis*, SSB) colony, generously provided by Prof. Yunhe Li from the Institute of Plant Protection, Chinese Academy of Agricultural Sciences, was used in this study. SSB larvae were reared on an artificial diet following Xue et al. [42] and kept in an insectary under a 12 h/12 h day/night cycle, with the temperature at 25–27 °C, and a relative humidity of 70–80%. Thirty third-instar larvae (each weighing approximately 20 mg) were fixed on the main stems of 30-d-old rice plants (one larva per plant) using a plastic tube (D × L: 3 cm × 6 cm) sealed at both ends with cotton (SSB). The corresponding control plants were caged in the same manner (Control). After 3 days, the stem tissues around the feeding sites of twenty infested plants with similar damage and the corresponding stem tissues of twenty control plants were sampled. The sampled stem tissues from five plants were pooled together for a single replicate and stored at −80 °C after flash freezing in liquid nitrogen for PA quantification and gene expression analysis.

### 4.3. PA Quantification

Frozen stem samples (approximately 0.5 g) were ground to a fine powder, and then homogenized in 5 mL of 5% perchloric acid (HClO_4_). The samples were extracted on a shaker for 24 h at room temperature. After centrifugation, the extracted supernatant was used to measure the free-type PAs. The PA concentration was analyzed by high-performance liquid chromatography (HPLC: LC-10A, Shimadzu, Kyoto, Japan) after the derivatization of PAs with benzoyl chloride, and 5 nmol 1,6-diaminohexane was used as an internal standard according to Chen et al. [43]. Each treatment had four replicates.

### 4.4. Gene Expression Analysis

For gene expression analysis, frozen stem samples (approximately 0.1 g) were used for RNA extraction. The gene expression analysis were conducted using a quantitative RT-PCR as previously described by Chen et al. [44], with Actin7 and EF1 as internal reference genes. The primer specificity was validated through and verified by a melt curve analysis. The 2^−ΔΔCt^ method was used for the relative expression calculation. As similar results were obtained using both reference genes, only the results based on Actin1 are presented. Each treatment had four replicates. The primers used to quantify gene expression levels are listed in Table 1.

### 4.5. Oral Secretion Treatment

Third-instar larvae were used for the collecting of oral secretions. The collected oral secretions were diluted 1:9 (*v*/*v*) with sterile distilled water. Each rice plant was mechanically wounded with a 0.5 cm diameter hole in the main stem using a puncher and 20 μL of diluted OS was applied at each wounded site (OS). Control plants were wounded and treated with 20 μL of sterile water (Control). The stem tissues around the wounded sites were harvested 24 h later for gene expression analysis.

### 4.6. Exogenous Application of Put and D-Arginine

Thirty-day-old uniform rice plants were subjected to five different treatments by applying PA or D-arginine in the nutrient solution: Mock (blank group without any treatment), Put (0.5 mM), Spd (0.5 mM), Spm (0.5 mM), and D-arginine (1 mM). At the fourth day of treatment, half the plants were subjected to SSB larval infestation as described above. After three days of infestation, the larvae were collected and weighed on an electronic balance (0.1 mg, ATX224, Shimadzu, Kyoto, Japan). The stem tissues around the feeding sites were sampled and stored at −80 °C after being frozen in liquid nitrogen for enzyme activity assays and PI analysis.

### 4.7. Exogenous Application of Put and DFMO

Third-instar larvae (each weighing approximately 20 mg) were fed on an artificial diet supplied with Put (0.01 mg·g^−1^) or DFMO (1 mg·g^−1^). A normal artificial diet without any treatment was used as the blank control (Mock). After three days of incubation, the larvae were weighed and the weight gain percentage was calculated. There were thirty replicates in each treatment.

### 4.8. Enzyme Activity Assays and PI Analysis

Frozen stem samples (approximately 0.5 g) were ground to a fine powder and then homogenized in 0.05 M phosphate buffer containing 1% (*w*/*v*) polyvinylpyrrolidone (PVP). After centrifugation with 12,000× *g* for 15 min at 4 °C, the extracted supernatant was used for enzyme assays. Peroxidase (POD) activity was analyzed using a colorimetric assay by monitoring the change of absorption at 420 nm due to guaiacol oxidation. Polyphenol oxidase (PPO) activity was determined using catechol as substrate according to the method of Ye et al. [38]. The accumulation of proteinase inhibitor was measured using the classical radial immunodiffusion assay. There were four replicates in each treatment.

### 4.9. Statistical Analysis

Statistical analysis was performed using the SPSS statistics software (Version 19.0 for Windows, SPSS, Chicago, IL, USA). The data were subjected to analysis of variance (ANOVA) with the least significant differences (LSD) post hoc test, and a *p* < 0.05 was considered to be significant.

## Figures and Tables

**Table 1:** Primers used in the real-time quantitative PCR experiment.

Gene	Accession Number	Primer
*ADC1*	LOC_Os06g04070	F: 5′-AGCTCCTGCACTTCCACATT-3′ R: 5′-CAAGCTGTATGCCACGGACA-3′
*ADC2*	LOC_Os04g01690	F: 5′-CCTACCGTGACAGAAGAAAGGA-3′ R: 5′-CACCCGAGGATGTTGTACACT-3′
*ODC1*	LOC_Os09g37120	F: 5′-CCATCTCCATCCCACGCTA-3′ R: 5′-CACGTTGCTAGTGTGTTTGGG-3′
*SAMDC2*	LOC_Os02g39795	F: 5′-CGAGCTGTCCAACAAGGACT-3′ R: 5′-TCACAGCAGCAAGTGGCATA-3′
*Actin7*	LOC_Os11g06390	F: 5′-ACTGTCCCCATCTA TGAAGGA-3′ R: 5′-CTGCTGGAATGTGCTGAGAGA-3′

## Data Availability

Not applicable.

## Supplementary Materials

The following supporting information can be downloaded at: https://www.mdpi.com/article/10.3390/plants12183249/s1, Table S1: Reads count and fold change of polyamine biosynthesis genes in previously published RNA-seq data collected from the rice plants infested with third-instar *Chilo suppressalis* (SSB) for 24 h.
