# Supplementary material for: Herbivory by Striped Stem Borer Triggers Polyamine Accumulation in Host Rice Plants to Promote Its Larval Growth
Source: Plants (Basel). 2023 Sep 13;12(18):3249. doi: 10.3390/plants12183249 (PMC10534419; doi:10.3390/plants12183249)
Supplement: Supplementary file 1 [file plants-12-03249-s001.zip › plants-2596306-supplementary.pdf]

Supplementary Table S1. Reads count and fold change of polyamine biosynthesis genes in previously published RNA-seq data collected from the rice plants infested with third instar *Chilo suppressalis* (SSB) for 24 h.

Gene name	Accession number	SSB_readcount	Control_readcount	Fold Change	*p* value
OsADC1	Os06g04070	10830.99	1323.10	8.19	0.00
OsADC2	Os04g01690	1251.26	54.37	23.01	0.00
OsADC3	Os08g33620	2.79	3.46	0.80	0.88
OsODC1	Os09g37120	1828.88	820.38	2.23	0.00
OsODC2	Os04g04980	1.60	1.86	0.86	0.88
OsODC3	Os02g28110	267.05	297.48	0.90	0.63
OsODC4	Os03g45230	19.19	23.27	0.82	0.66
OsSAMDC1	Os04g42095	41766.05	35882.97	1.16	0.14
OsSAMDC2	Os02g39795	21078.61	6266.70	3.36	0.00
OsSAMDC3	Os05g04990	N.D.	N.D.	--	--
OsSAMDC4	Os09g25625	594.02	504.19	1.18	0.30
OsSAMDC5	Os09g24600	N.D.	N.D.	--	--
OsSAMDC6	Os05g13480	N.D.	N.D.	--	--

N.D.: Not detected.
